# Performance estimation of optical skin probe in short wavelength infrared spectroscopy based on Monte-Carlo simulation

**DOI:** 10.1038/s41598-022-23251-4

**Published:** 2022-11-22

**Authors:** June-Young Lee, Sungmo Ahn, Sung Hyun Nam

**Affiliations:** Samsung Advanced Institute of Technology, 130 Samsung-Ro, Yeongtong-Gu, Suwon-Si, Gyeonggi-Do Korea

**Keywords:** Health care, Engineering, Optics and photonics

## Abstract

Optical throughput and optical path length are key parameters to obtain high signal to noise ratio and sensor sensitivity for the detection of skin tissue components based on short wavelength infrared (SWIR) spectroscopy. These parameters should be taken into account at the stage of optical system design. We aim to develop a method to estimate the optical efficiency and the effective water path length of a newly designed SWIR spectroscopy skin measurement system using Monte-Carlo photon migration simulation. To estimate the optical efficiency and the effective water path length, we investigated the characteristics of Monte-Carlo photon migration simulation utilizing one layered simple skin model. Simulation of photon transport in skin was conducted for transmission, transflection, and reflection optical configurations in both first overtone (1540 ~ 1820 nm) and combination (2040 ~ 2380 nm) wavelength ranges. Experimental measurement of skin spectrum was done using Fourier transform infrared spectroscopy based system to validate the estimation performance. Overall, the simulated results for optical efficiency and effective water path length are in good agreements with the experimental measurements, which shows the suggested method can be used as a means for the performance estimation and the design optimization of various in-vivo SWIR spectroscopic system.

## Introduction

In-vivo optical measurement based on short-wavelength infrared spectroscopy has been widely used for noninvasive biomedical applications^1–5^. The amount of target components in skin can be quantified by measuring the spectral absorbance of the skin. Major skin components including water, protein and lipids or metabolites such as glucose and lactate have their characteristic absorption spectra in SWIR wavelength range^6–8^. Thus, SWIR spectroscopy has been applied to measuring the changes in the components of skin due to aging^7^, analyzing secondary structural changes in skin protein^9^ or sensing the concentration of glucose or lactate in skin tissue^3,4,10–12^.

When the SWIR spectroscopy is used to measure physiological components in skin, the following two aspects should be considered carefully. First, if the amount of the target component is minute compared to those of whole background skin components, securing sufficient signal to noise ratio (SNR) is essential. For example, the SWIR absorbance of glucose or lactate in skin is in the range of 10^−5^ ~ 10^−3^ absorbance unit (AU), while the total absorbance of skin is typically several 10^−1^ ~ 1 AU in millimeter scale path length. Thus, optical efficiency for obtaining enough SNR should be accurately estimated at the stage of measurement system design. Second, estimating the skin water path length precisely is also as important since it determines the sensor sensitivity necessary for successful in-vivo measurement, where the physiological components of interest such as metabolites or nutrients are dissolved in extracellular or intracellular fluid^13^.

Since Wilson et al. firstly implemented^14^ and Prahl et al. subsequently developed Monte Carlo (MC) method for simulating light propagation in tissue by applying variance reduction technique^15^, MC photon migration simulation has been a standard approach for photon transport calculation in turbid media^16–18^. The probabilistic interpretation of the radiative transfer equation (RTE), which theoretically describes the photon movement in turbid media, leads to a statistical MC photon migration process^19,20^. In practice, MC-based photon migration simulation has been used in the research on in-vivo optical measurement and noninvasive biomedical diagnosis technology to investigate the distribution of illuminated photons in biological tissue^12,21–30^. A lot of study on modeling sampling volume and spectra in relation to fiber-optics probes have been conducted^31–33^, voxelized MC^18^ or GPU acceleration^34–36^ were developed to simulate millions of photons in a matter of seconds, and machine learning combined MC methods were utilized to reduce time-consuming calculations^37–41^, yet sparse research has been performed in SWIR wavelength range.

In this study, we propose a MC based optical skin simulation method to estimate the performance of a newly designed SWIR skin measurement system in terms of both the optical efficiency and the effective skin water path length characteristics. Precise comparison between various optical configurations was done using this method. In the following sections, optical skin simulation using MC photon migration method is performed for transmission, transflection and reflection skin measurement modes, as these three modes can cover all the possible optical configurations for skin measurement. To verify the feasibility of the method, the simulation results are compared with the experimental skin measurement results in each configuration.

## Methods

### Optical configurations

**Figure 1:**
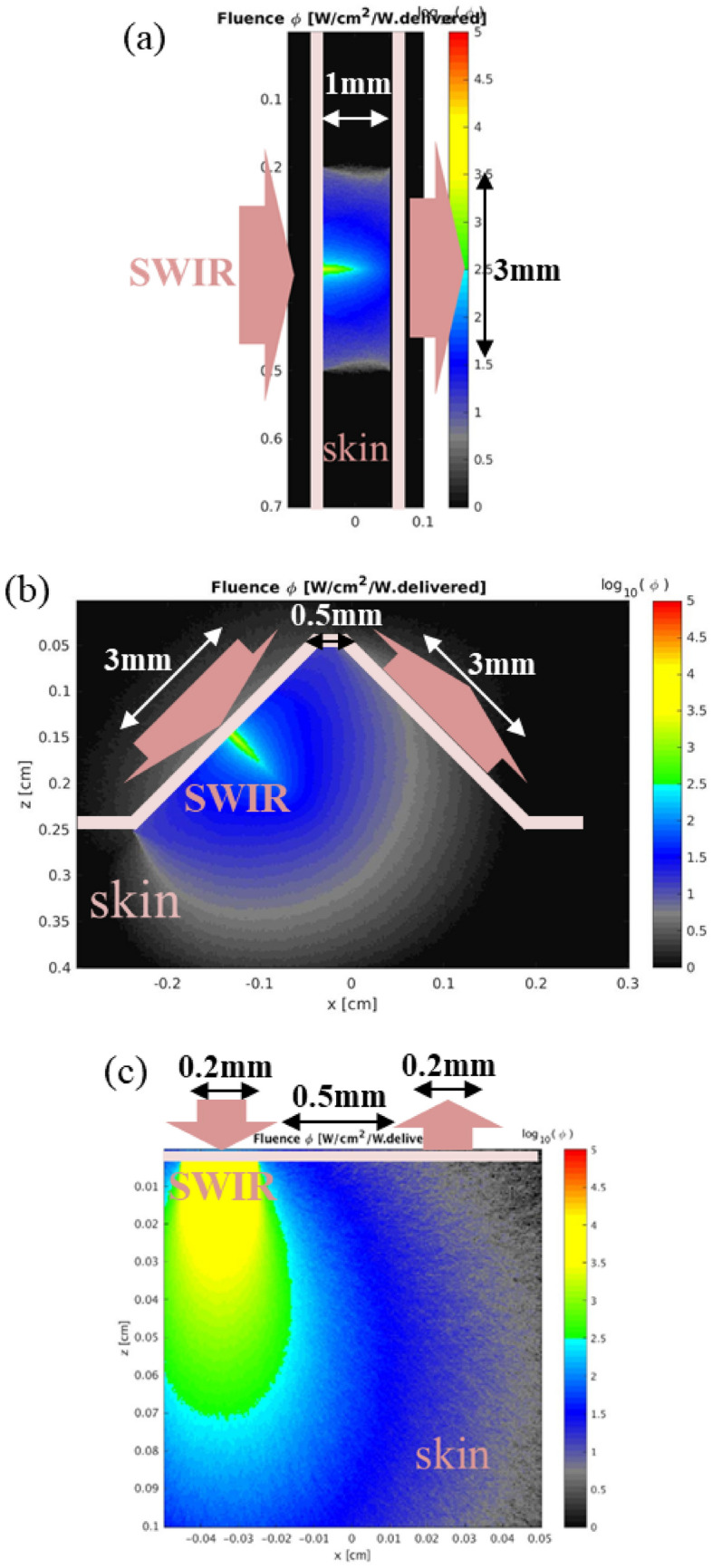
Skin-SWIR optical interaction simulated by MC method for (**a**) transmission, (**b**) transflection and (**c**) reflection optical configurations.

Figure 1 shows three representative optical configurations for skin measurement investigated in this study. For the transmission and the transflection configurations, sapphire fiber optic waveguides with 3 mm diameter are attached to skin for both illumination and collection of SWIR light, while low-OH optical fibers with 0.2 mm diameter are in contact with skin to allow physical space for input and output channels within acceptable optical path length in reflection configuration. Separation length between the illumination and the collection waveguides for each configuration was set respectively to obtain approximately 1 mm average photon travel length in skin. Thickness of statum corneum and epidermis layers is known to be 10 ~ 20 um and 50 ~ 80 um^42^, respectively, except heavily used parts of the body like palms of the hands or soles of the feet where the epidermis layer is thicker than 1.5 mm. Thus, the illuminated photons of the optical system used in this study were designed to travel mainly in dermis layer that has abundant extracellular fluid. Two fiber-optic interfaces are located across a 1 mm thick folded skin for the transmission configuration. For the transflection configuration, the illumination and the collection optical fibers are separated by 0.5 mm and positioned in 90-degree geometry. The separation between the waveguides is set to be 0.5 mm for the reflection configuration.

### Simulation parameters

**Figure 2:**
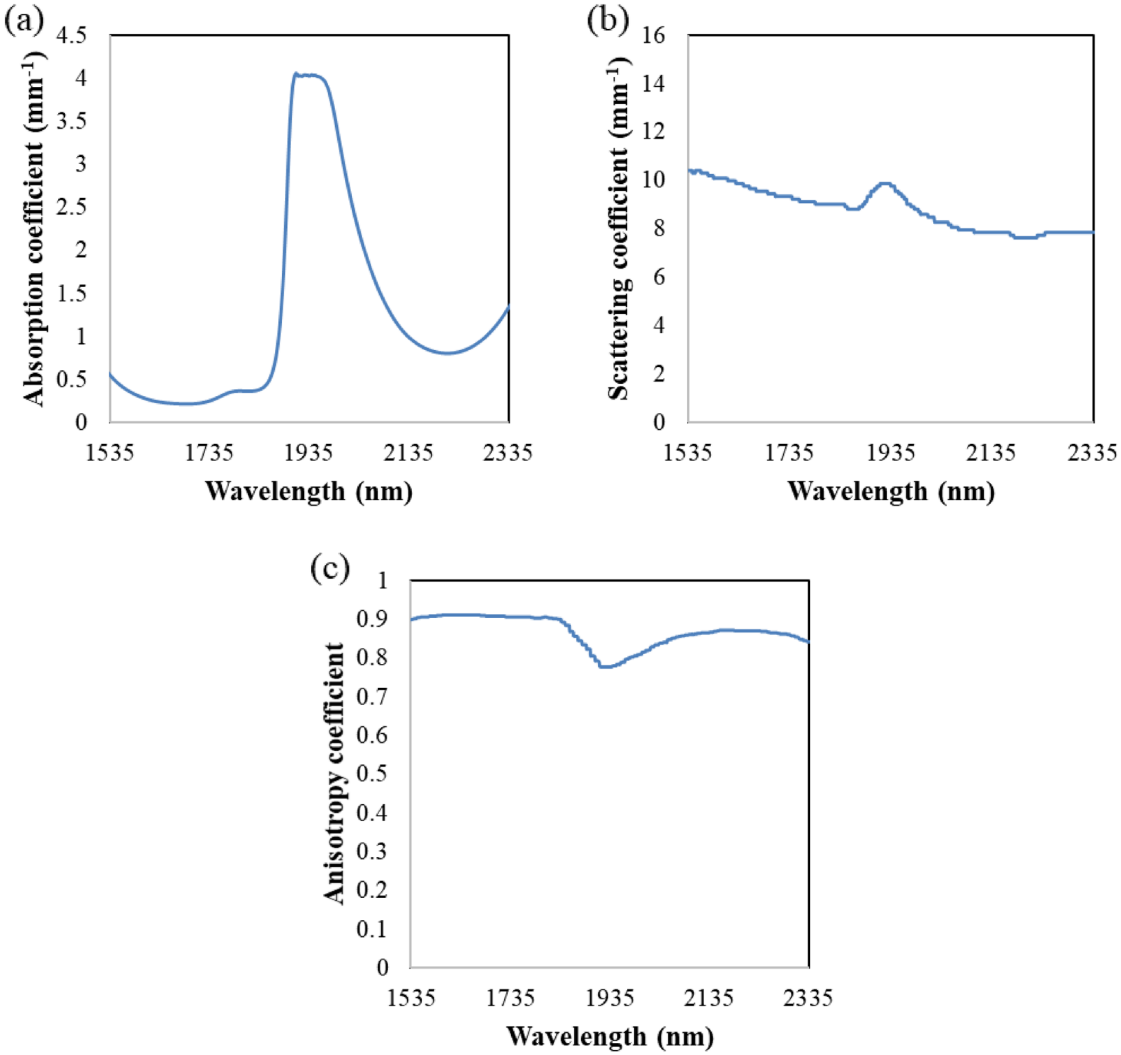
Optical coefficients for the simulated skin layer (**a**) absorption, (**b**) scattering and (**c**) anisotropy coefficient.

The basic MC simulation algorithm from the Oregon Medical Laser Center^18^ was modified for this study. Although the numerical simulation was performed by MATLAB (MathWorks, USA), commercial software like Zemax (Ansys, USA) or TracePro (Lambda Research Corporation, USA) are also available for this MC simulation^43–45^. Illuminating 100,000 SWIR photon packets was adequate for the transmission and the transflection configurations while at least 10,000,000 SWIR photon packets were required for the reflection configuration due to the nature of extremely low light throughput. As aforementioned, the optical design of the skin measurement systems used in this study let the illuminated photons travel mainly in dermis layer. Thus, unlike previous studies which used multiple layers as skin model^31,46,47^, the skin matrix in this simulation was assumed to be one homogeneous layer to alleviate the modeling complexity without losing generality of conclusions. Likewise, as shown in Fig. 2a, water absorptivity at 37 ℃ was used to represent the absorption coefficient of the one layered medium where the most significant absorber in skin is known to be water. The scattering and anisotropy coefficients for this simulation are shown in Fig. 2b,c, which refer to the experimental results by Roggan et al.^48^. The refractive index of skin is assumed to be 1.4. The first overtone (O, 1540 ~ 1820 nm) and the combination (C, 2040 ~ 2380 nm) regions were selected for the wavelength ranges of interest, as these windows have specific spectroscopic features for various physiological molecules^49^. Besides, SWIR absorption features of physiological molecules become significantly weaker and broader as the wavelength decreases, thereby greatly reducing the analytical utility of the shorter wavelength region in terms of molecular vibrational information. Thus, the first overtone and the combination regions were chosen for this study. The spectral resolution of this simulation was chosen to be 5 nm, which required simulations of approximately 60 and 70 wavelength points for the overtone and the combination regions, respectively. The simulations were conducted in Samsung Electronics supercomputing system with 65,212 CPU cores.

The number of detected photons and the travel length of each photon at each wavelength were calculated in the skin matrix placed between the optical probes. To obtain reference spectra, detected photon numbers were calculated with empty space between the probes for the transmission and the transflection configurations, which imitates the experiment. For the reflection configuration, reference spectra were calculated with a 99% Spectralon (Labsphere, USA), which is a standard diffuse reflector, attached to the probe. To meet the reflectance specification (0.95 ~ 0.99) provided by the manufacturer, absorption, scattering and anisotropy coefficients of the Spectralon were chosen to be 0.0001 mm^−1^, 8000 mm^−1^ and 0.1, respectively.

### Optical instrumentation and skin measurement

**Figure 3:**
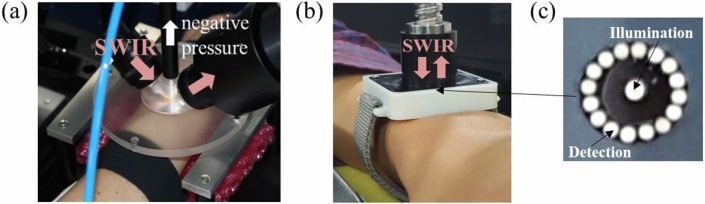
SWIR Skin measurement system with a transflection configuration (**a**) and a reflection configuration (**b**), enlarged bottom view of the reflection skin probe composed of 1 illumination and 16 detection optical fibers (**c**).

Experiments were performed on the wrist skin with the transflection and the reflection configurations. Institutional Review Board in Samsung Medical Center approved this study (Protocol number: 20171103001). The study was performed in accordance with the relevant guidelines and regulations, and informed consent was obtained from the subject before the experiment. A bench-top system was implemented by using a conventional FTIR (Fourier transform infrared) spectrometer (VERTEX 80, Bruker, Germany) with a thermoelectrically cooled and extended InGaAs photo diode and skin interface probes. The detector element had a spectral cutoff of 2.6 um (3846 cm^−1^) and was operated at – 40 ℃. 150 W tungsten-halogen lamp was used to provide steady-state broadband SWIR light. Detected SWIR signal was amplified up to 100 times to reduce electrical noise. Each spectrum had a resolution of 16 cm^−1^. Optical power of the illuminated and the detected light was measured using an optical power meter (2936-R, Newport, USA). For the transflection configuration, the skin interface probe was machined out of acrylic material to provide a conical-shaped cavity into which the skin tissue was drawn up by a vacuum as shown in Fig. 3a. For the reflection configuration shown in Fig. 3b, 16 detection optical fibers surrounded an illumination optical fiber with 0.5 mm separation as shown in Fig. 3c. 3 mm diameter sapphire rods having 1.44 numerical aperture (NA) were used for the transflection configuration. 200 um diameter low OH fused silica fibers having 0.22 NA were used for the reflection configuration. Fiber bundle for the reflection configuration was custom-made (Green Optics, Korea). Sapphire rods were also fabricated from the same manufacturer. While sapphire rod had no cladding material, general cladding material (fluorine doped glass) was used for the fused silica optical fiber. The reference for the calculation of absorbance spectra were measured with air in the interface for the transflection configuration and with a 99% Spectralon attached to the probe for the reflection configuration.

## Results and discussion

### Optical efficiency

The simulated photon distribution in the skin matrix for each optical configuration is shown in Fig. 1. There are relatively higher fluence rate in the central area of the profile for transmission and transfection configurations in Fig. 1a,b. In the experimental system, the diameter of illumination light, which was modulated and went out of FTIR, was around 50 mm. To put this light into 3 mm diameter optical fiber, focusing lens was used. This lens optics was included in the simulation, and caused higher fluence rate in the central area near the illuminated skin surface.

The simulated optical efficiency is defined as the number of detected photons divided by the number of illuminated photons. The optical efficiency is also measured by taking the ratio of the illuminated and the detected light power in the experiments. The simulation result shows ranges of the optical efficiency along the wavelength change. This comes from the fact that the optical coefficients of skin vary with the wavelength. In the experiment, the optical power was measured with a band pass filter of 400 nm bandwidth. Thus, the experimental result of the optical efficiency is a representative value within the wavelength band of the filter. Table 1 compares the simulation with the experimental results of the optical efficiency for each configuration and wavelength range. Although the experimental measurement has a tendency of slightly lower optical efficiency than the simulation, the comparison shows that both results are within the same order of magnitude. Optical power measurement at each wavelength or simulation exploiting multi layered skin model can be considered to investigate further improvement of the estimation accuracy in the future.

**Table 1:** Optical efficiency comparison between simulation and experiment for each optical configuration.

Optical efficiency	Transmission	Transflection	Reflection
Simulation	10^−1^ ~ 3 × 10^−1^ (O), 1 × 10^−3^ ~ 5 × 10^−2^ (C)	10^−3^ ~ 3 × 10^−2^ (O), 10^−4^ ~ 3 × 10^−3^ (C)	10^−4^ ~ 2 × 10^−4^ (O), 3 × 10^−6^ ~ 5 × 10^−6^ (C)
Experiment	N/A	10^−3^ (O), 10^−4^ (C)	10^−4^ (O), 10^−6^ (C)

Several orders of magnitude difference in optical efficiency is apparent between the measurement configurations. Transmission configuration has approximately ten times larger peak optical efficiency than transflection configuration. Likewise, transflection configuration has approximately several tens to hundred times larger peak optical efficiency than reflection configuration. Optical efficiency differs between wavelength ranges also. In the first overtone wavelength range, the optical efficiency is ten to hundred times larger than the combination wavelength range. This difference between wavelength ranges is mainly from the characteristics of relatively high water absorption coefficient in the combination wavelength region.

### Photon travel length

Photon travel length is defined as the trajectory length of a photon from the incident point to the exit point of a skin matrix. Due to random multiple scattering process, there are various trajectories of photons traveling in the skin matrix. During the simulation, each photon has record of its traveling path. After selecting photons that arrived at the detector, the travel length of each photon can be calculated with its record.

**Figure 4:**
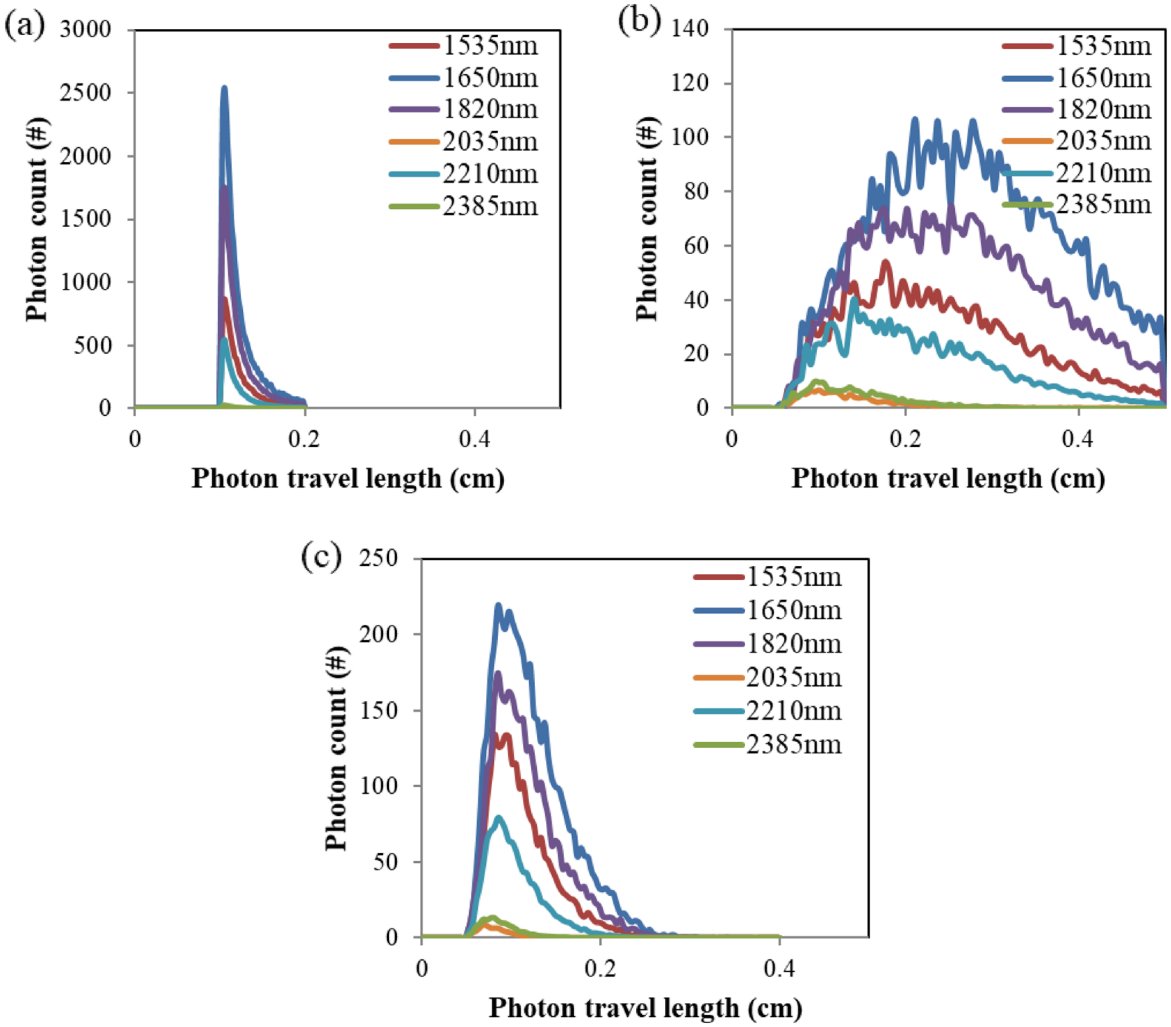
Distribution of the photon travel length at each wavelength for (**a**) transmission, (**b**) transflection and (**c**) reflection optical configurations.

Simulated distributions of the photon travel length at various wavelengths for each optical configuration are compared using histogram plots in Fig. 4. Wavelength dependency of the photon travel length distribution results from difference in values of absorption, scattering and anisotropy coefficient for each wavelength. Generally, more photons in the first overtone range survive and arrive at the detector due to low absorptivity compared to photons in the combination range. Relatively narrow distribution of the photon travel length for transmission mode in Fig. 4a shows that forward scattering is dominant in skin media. As shown in Fig. 4b, the transflection mode exhibits the broadest distribution of travel length, which matches well with its conical shaped geometry where various path lengths exist between the illuminating and the detecting waveguides.

**Figure 5:**
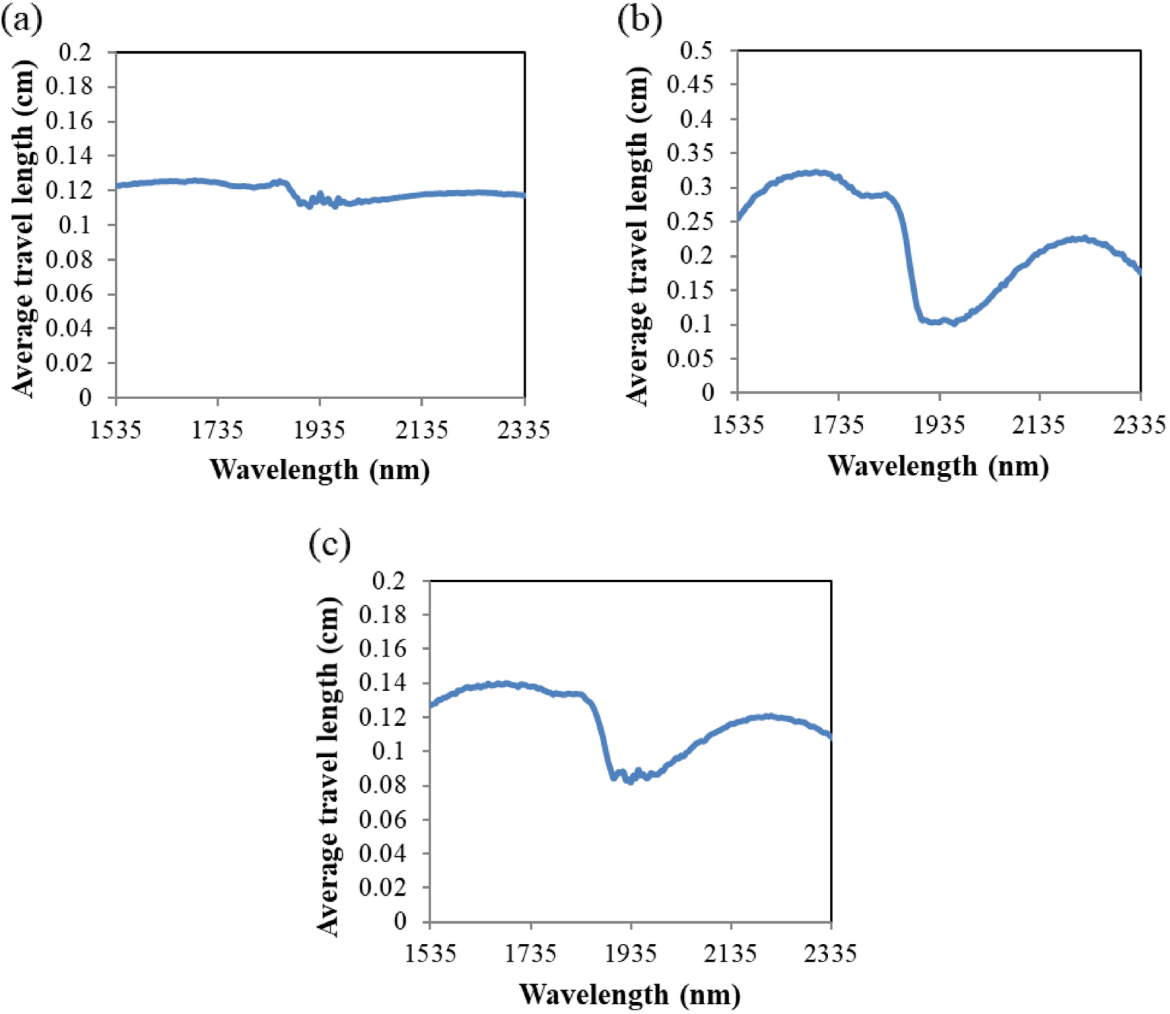
Average photon travel length along the wavelength for (**a**) transmission, (**b**) transflection and (**c**) reflection optical configurations.

Average values of the photon travel length for various wavelengths are shown in Fig. 5. The transmission configuration shows relatively leveled photon travel length along the wavelength change. In contrast, wide ranges of average photon travel length exist in the transflection and the reflection configurations. The wavelength profiles of these two configurations are similar to that of anisotropy coefficient. For these two configurations, the photons need to be scattered and change its direction to arrive at the detector, where higher anisotropy coefficient results in smaller change in photon direction and results in longer trajectory between the source and the detector.

### Absorbance spectra

**Figure 6:**
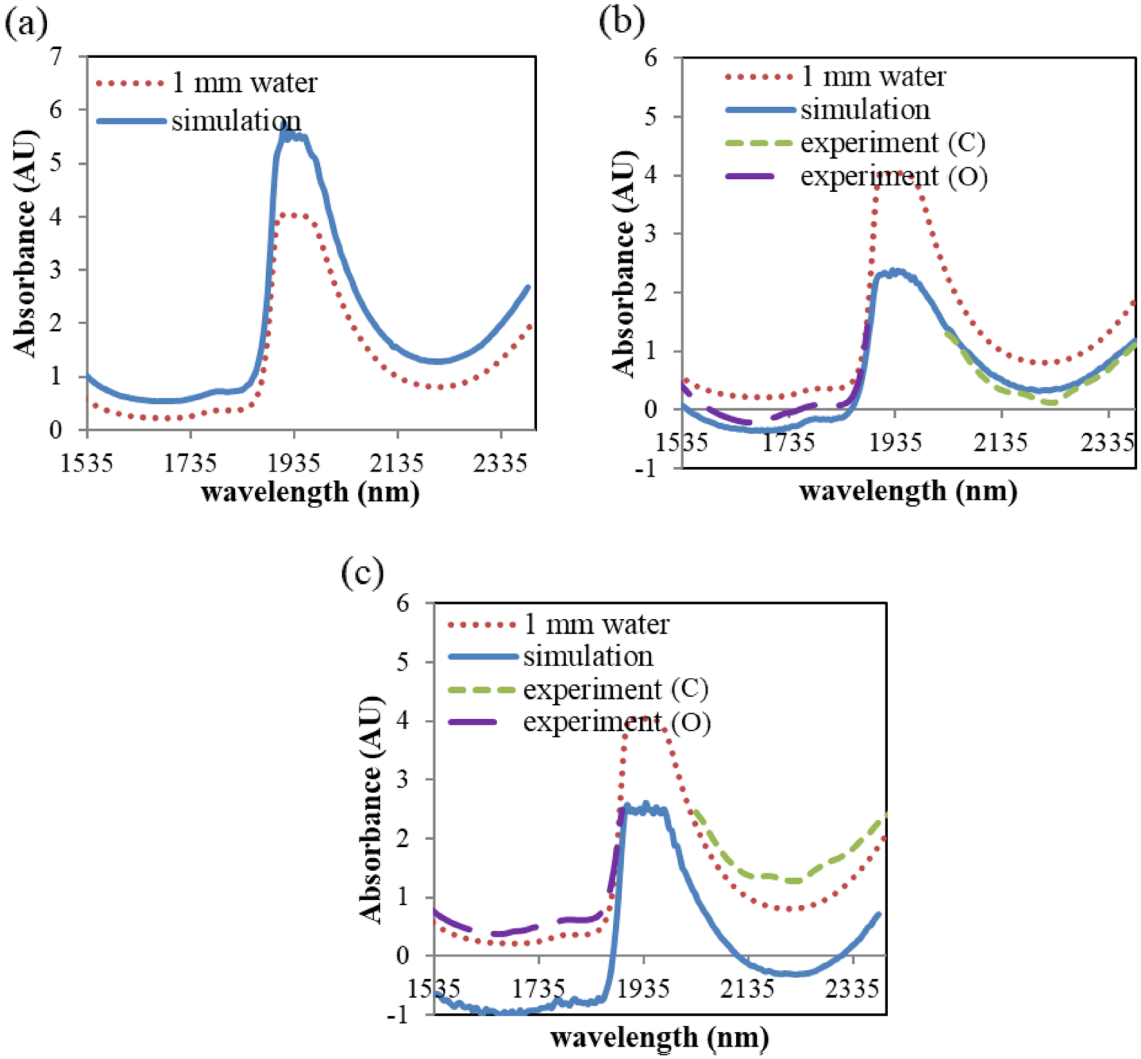
Simulated and measured skin spectra for (**a**) transmission, (**b**) transflection and (**c**) reflection configurations compared with water spectrum.

The simulated skin absorbance spectra for each optical configuration are compared with the measured skin spectra and the water absorption coefficient in Fig. 6. The simulated skin spectrum for the transmission configuration shows larger absorbance than the water absorption spectrum in Fig. 6a. Although the separation between the source and the detector is set to be 1 mm, a number of photons travel longer than 1 mm path due to the scattering in skin. The photons experience more absorption and as a result, the absorbance is larger than the absorbance of water in 1 mm thick cuvette.

In the transflection configuration, the absorbance is lower than that of 1 mm thick water. Due to the 90-degree angle configuration between the source and the detection waveguide, small amount of the illuminated photons can be detected in air, while more photons in skin can reach the detection waveguide owing to the scattering characteristics of the skin. The output light intensity for the air reference is smaller than that of the skin. Thus, the absorbance of skin in the first overtone range can take negative values. Negative absorbance can be prevented if the air reference is defined as just the mount of illuminated photons. This negative absorbance, however, doesn’t have any influence on the calculation of the optical efficiency or the effective water path length. Optical efficiency takes only the illuminated photons as a reference, and negative absorbance can be compensated by offset correction during the effective water path length calculation.

For the case of the reflection configuration, the measured skin spectrum shows larger absorbance than water, while the simulated skin spectrum is far lower than the water absorbance curve and has negative value at some wavelength range. Diffuse reflection at the rough surface of the actual Spectralon was not taken into account in this simulation^50^. The surface of the Spectralon was set to be flat and mirror-like in the simulation. The majority of the photons were reflected back at the surface around the position of the illumination waveguide, and could not reach the detection waveguide which is separated from the illumination waveguide with a 0.2 mm gap. On the other hand, the actual surface of the Spectralon is rougher, so more diffusely reflected photons could have chances to arrive at the detection waveguide. The simulated model for the Spectralon has smaller reflected photons than the experimental result, which leads to constant offset difference between the simulated and the measured skin absorbance spectra. Similar to the characteristics of negative absorbance in the transflection configuration, this offset also doesn’t have any negative influence on the calculation of the optical efficiency or the effective water path length.

While the simulated skin spectra by using water absorption coefficient only has smooth line shapes, the measured skin spectra have a few small peaks especially in the combination wavelength range. These peaks near 2182, 2238, 2318 nm are owing to the absorption from other skin components such as protein and fat. The overall shape of the simulated skin spectra, however, is similar to the measured spectra as the major absorption component of the skin is water.

### Effective water path length

To quantify the concentration of target molecules dissolved in skin fluid by Beer’s law, the effective water path length needs to be calculated first from the measured spectra^3^. The effective water path length can be linearly regressed from the skin spectrum based on multiple scattering correction (MSC) method where the skin components’ absorptivities, constant and slope terms are used^3,51^ as the following equation:

1 $${\mathrm{A}}_{skin}={\mathrm{c}}_{water}{\mathrm{A}}_{water}+{\mathrm{c}}_{collagen}{\mathrm{A}}_{collagen}+{\mathrm{c}}_{keratin}{\mathrm{A}}_{keratin}+{\mathrm{c}}_{fat}{\mathrm{A}}_{fat}+{\mathrm{c}}_{offset}{\mathrm{A}}_{offset}+{\mathrm{c}}_{slope}{\mathrm{A}}_{slope}+\upvarepsilon ,$$

where ${\mathrm{A}}_{skin}$ represents the measured skin spectrum,${\mathrm{A}}_{water}$, ${\mathrm{A}}_{collagen}$, ${\mathrm{A}}_{keratin}$ and ${\mathrm{A}}_{fat}$ represent pure component absorption of water, collagen, keratin, and fat, respectively, ${\mathrm{A}}_{offset}$ and ${\mathrm{A}}_{slope}$ correspond to the offset and slope terms, respectively, ${\mathrm{c}}_{water}$, ${\mathrm{c}}_{collagen}$, ${\mathrm{c}}_{keratin}$, ${\mathrm{c}}_{fat}$, ${\mathrm{c}}_{offset}$ and ${\mathrm{c}}_{slope}$ represent regression coefficients from fitting the corresponding standard absorbance spectrum, and ε is residual spectrum. The regression coefficient of water (${\mathrm{c}}_{water}$) from this spectral fits indicates the amount of water within the optical path. This effective water path length is related to the relative difference of detected photon numbers between center and outer wavelengths of the overtone or the combination region. Water absorption is strongest near outer parts of each wavelength region (1500, 1800, 2000 and 2500 nm), thus the amount of detected photons is lowest near these wavelengths. Meanwhile, water absorption is weakest near the center wavelengths (1650 and 2250 nm) and the amount of detected photons is highest near these wavelength regions. Table 2 summarizes the regressed effective water path length from the simulated and the measured skin spectra in each optical configuration shown in Fig. 6.

**Table 2:** Effective water path length comparison between simulation and experiment for each optical configuration.

Effective water path length	Transmission	Transflection	Reflection
Simulation	1.33 mm (O), 1.28 mm (C)	1.25 mm (O), 0.82 mm (C)	1.12 mm (O), 0.97 mm (C)
Experiment	NA	1.80 mm (O), 0.85 mm (C)	1.20 mm (O), 0.93 mm (C)

The regressed effective water path length for transmission mode is 1.33 mm for the first overtone range and 1.28 mm for the combination range. These values are somewhat larger than the average photon travel length which is in the range of 1.21 ~ 1.25 mm for overtone and 1.17 ~ 1.19 mm for combination region. This difference seems to result from the distribution shape of photon travel length in Fig. 4, where the distribution shows a longer tail from the average value toward larger travel length numbers.

Comparison between the simulated and the measured water path length of the transflection and the reflection modes shows good consistency except for the first overtone range of transflection mode, where the simulated value (1.25 mm) is 31% smaller than the measured value (1.8 mm). The nature of wide photon travel length distribution of the transflection configuration in the first overtone region seems to influence this result. Unlike the other configurations, a large portion of photons travel longer than 2 mm in the first overtone wavelength region in the transflection configuration as shown in Fig. 4b. When the photons travel longer distance in skin, there exists more uncertainty in optical parameters used in simulation due to the skin inhomogeneity. The photons with wavelength near 1500 or 1800 nm experienced more extinction than simulated, which led to the discrepancy in absorbance between the simulation and the measurement. As a result, the measured absorbance in Fig. 4b shows relatively higher values than simulated absorbance near 1500 and 1800 nm. In the future, simulation exploiting multi layered skin model can be used to investigate further improvement of the estimation accuracy. For the other three cases, the simulated water path length matches well to the measured value with the error less than 6.7%.

### Selection of optimal skin probe design

Overall, the transmission configuration shows the largest optical efficiency and the smallest photon travel length variation along the wavelength range in use, which can provide the best performance from the spectroscopic point of view. However, the locations for in vivo measurement are limited to the earlobes or other folded skin for this configuration.

While the transflection configuration has intermediate optical performance and can be applied to measurement of general skin surfaces, pulling up the skin surface and maintaining the configuration shape may require complex mechanical parts such as vacuum pumping.

Probing principle of the reflection measurement is more convenient to use in obtaining spectra compared with the transmission or transflection measurements. The reflectance measurement can be applied to most of the skin surface. However, the reflectance configuration has extremely low light throughput compared to the other configurations. Thus, multi-optical fibers based skin probe providing large optical throughput or high power light sources such as super luminescent diodes (SLD) or tunable lasers may be better choices to acquire enough optical SNR.

Once the measurement site on skin is given, an optimal configuration could be determined. Then, precise performance estimation and optimization of the detailed skin probe design can be carried out using the simulation methodology in this study.

## Conclusion

We have investigated the key performance parameters of an in-vivo SWIR spectroscopic system, which are the optical efficiency, the effective skin water path length, and the spectral absorbance in three different configurations, by using MC based optical skin simulation. The results were tested by comparing them with the experimental skin measurement ones. The analysis presented in this study will be beneficial in design optimization of various in-vivo SWIR spectroscopic systems by providing the performance of the key design parameters. Our future work entails simulation using multi layered skin model for further enhancement of the estimation accuracy and comparison between simulation and experiments at multiple dimension parameters such as source-detector separation or fiber angle for the detailed design optimization based on the simulation methodology suggested in this study.

## Data Availability

The datasets used and/or analysed during the current study are available from the corresponding author on reasonable request.
